# Initial Assessment of a Comprehensive Digital Smoking Cessation Program That Incorporates a Mobile App, Breath Sensor, and Coaching: Cohort Study

**DOI:** 10.2196/12609

**Published:** 2019-02-04

**Authors:** Jennifer D Marler, Craig A Fujii, David S Utley, Lydia J Tesfamariam, Joseph A Galanko, Heather Patrick

**Affiliations:** 1 Carrot Inc Redwood City, CA United States; 2 Biostatistics Core for the Center for Gastrointestinal Biology and Disease and the biostatistician for the Clinical Nutrition Research Center Department of Medicine, Division of Gastroenterology and Hepatology University of North Carolina at Chapel Hill Chapel Hill, NC United States

**Keywords:** smoking cessation, mobile applications, health promotion, cell phone

## Abstract

**Background:**

Cigarette smoking is the leading cause of preventable morbidity and mortality, excess health care expenditure, and lost work productivity. Otherwise effective evidence-based treatments have had limited success owing to challenges with access, engagement, and scale. Pivot is a comprehensive digital smoking cessation program that incorporates a Food and Drug Administration–cleared carbon monoxide breath sensor, smartphone app, and text-based human coaching.

**Objective:**

This initial evaluation of Pivot aimed to assess participant engagement, changes in attitudes toward quitting, and changes in smoking behavior.

**Methods:**

US cigarette smokers aged 18 to 65 years who smoked ≥5 cigarettes per day (CPD) were recruited online. Participants completed a screening call, electronic informed consent, registration, and onboarding before beginning Pivot. Pivot includes 5 sequential stages (Explore, Build, Mobilize, Quit, and Secure), taking 14.5 to 18.5 weeks to complete. Data were collected via app and online questionnaires. Outcomes included engagement and retention (ie, weeks of active engagement and Pivot stage progression); attitudes toward quitting (ie, quit readiness, quit confidence, and expected difficulty maintaining quit); and smoking behavior (ie, quit attempts, cigarette reduction, and abstinence (7- and 30-day point prevalence abstinence [PPA]).

**Results:**

A total of 319 participants completed onboarding (intention-to-treat [ITT] sample); 272/319 participants (85.3%) completed the end-of-Pivot questionnaire (study completer sample). Most (212/319, 66.5%) were not ready to quit in the next 30 days at baseline. On average, participants actively engaged in the program for a mean 12.4 (SD 7.1) weeks. Pivot stage completion rates were Explore: 88.7% (283/319), Build: 57.4% (183/319), Mobilize: 43.6% (139/319), Quit: 41.1% (131/319), and Secure: 39.5% (126/319). Repeated measures linear mixed model analyses demonstrated positive changes in attitudes from baseline to Mobilize (pre-Quit): increased confidence to quit (4.2 to 7.4, *P*<.001) and decreased expected difficulty maintaining quit (3.1 to 6.8, *P*<.001).

The quit attempt rate (ie, those making ≥1 quit attempt lasting ≥1 day) was 79.4% (216/272, completer). At the end of Pivot, 7-day PPA rates were 32.0% (102/319, ITT) and 37.5% (102/272, completer); 30-day PPA rates were 27.6% (88/319, ITT) and 32.4% (88/272, completer). Moreover, 30-day PPA rates were comparable among those ready and not ready to quit in the next 30 days at baseline. Of those not achieving abstinence, 25.9% (44/170, completer) achieved ≥50% reduction in CPD by study end.

**Conclusions:**

This study evaluated Pivot’s initial performance with comparable quit rates among those ready and not ready to quit in the next 30 days at entry. The present data, considered with the program’s accessibility, innovation, evidence-based foundation, and design for all smokers, suggest Pivot has the potential to address limitations of reach and scale and thereby advance smoking cessation efforts.

**Trial Registration:**

ClinicalTrials.gov NCT03295643; https://clinicaltrials.gov/ct2/show/NCT03295643 (Archived by WebCite at http://www.webcitation.org/75TiNe6BE).

## Introduction

### Background

In the United States, cigarette smoking is responsible for 480,000 deaths annually, and more than 16 million individuals live with a smoking-related illness [1]. Meaningful gains have been made in smoking cessation over the last decade, with the prevalence of cigarette smoking among US adults decreasing from 20.9% in 2005 to 14.8% in 2017 [2,3]. Despite this progress, smoking remains the leading cause of preventable morbidity and mortality.

Approximately 70% of adult smokers want to quit, and a little over half attempt to quit each year. Yet, quit rates are low, with less than 1 in 10 quit attempts being successful. This is related, in part, to underutilization of proven treatments, which include behavioral counseling and pharmacotherapy [4]. Use of behavioral counseling or cessation medications approximately doubles quit rates with combined use being more effective than either alone [4-6]. Even so, fewer than a third of those making a quit attempt use any evidence-based cessation methods, and less than 5% use the optimal approach of combination counseling and pharmacotherapy [7,8]. There are multiple causes of this underutilization, including health care access, transportation challenges, time constraints, convenience, awareness of service availability, desire to quit unassisted, and costs. State quitlines overcome many of these barriers but, on average, reach only about 1% of smokers annually [9]. Overall, the end result is that otherwise effective treatments suffer limited population reach, which ultimately undermines their impact on smoking cessation.

Acknowledging the limitations of current treatment methods, particularly relating to reach and scalability, the rise of smartphone ownership presents an appealing medium for smoking cessation treatment. As of 2017, 77% of Americans owned a smartphone, with majority-level ownership rates across various demographic and socioeconomic characteristics (eg, ≥75% ownership rates for varying ethnic and racial groups and ≥67% for all income groups) [10]. Research suggests smokers are actively using smartphones in quit attempts, with 1 study reporting an average of 779,400 downloads of Android platform–based smoking cessation apps per month worldwide [11]. Another study reported that a little over half of the smokers had downloaded smoking cessation apps in the past and, of these, three-quarters had made quit attempts using an app [12].

Despite this interest and use, quality among smoking cessation apps is disparate. Haskins et al evaluated 158 peer-reviewed articles addressing mobile apps for smoking cessation and identified 177 unique apps relevant to smoking cessation in the App Store for iPhone and 139 in Google Play for Android. They ultimately identified only 6 apps with some level of scientific support in the peer-reviewed literature, only 3 of which were available in the app stores [13]. Although, to date, there has been limited availability of evidence-based mobile smoking cessation programs, recent assessments of such programs have yielded encouraging results. Bricker et al evaluated a smoking cessation app focused on Acceptance and Commitment Therapy (ACT) in 99 adult smokers. At 2-month follow-up, 84% of study completers were satisfied with the program, 21% had achieved 7-day point prevalence abstinence (PPA), and 11% had achieved 30-day PPA [14]. In 416 smokers, Iacoviello et al assessed a smoking cessation app designed to deliver the essential features of the United States Clinical Practice Guideline (USCPG) for treating tobacco use and dependence [4]. They reported a 7-day PPA of 45.2% and 30-day PPA of 26.2% in an intent-to-treat (ITT) analysis [15].

Building on these efforts, Pivot is a comprehensive digital smoking cessation program that brings together multiple evidence-based components into a seamless solution. In addition to delivering the USCPG through a multiphase mobile app, Pivot includes the first Food and Drug Administration (FDA)-cleared personal carbon monoxide (CO) breath sensor, dedicated human coaching delivered through in-app text messaging, and a program designed for individuals with varying levels of readiness to quit. In line with wearable devices, the CO breath sensor provides real-time personal biometric data to users. This leverages the findings of several published studies [16-19] as well as expert opinion [20,21], which suggest that personal CO breath sample data can be educational and motivational and may lead to changes in attitudes toward quitting and smoking behavior. To that end, the CO breath sensor is incorporated in the Pivot program as an engagement tool, with the intention that users will find their expired CO values informative and motivational. Pivot coaching incorporates evidence-based principles of smoking cessation treatment combined with innovation through digital delivery. Continuity of care is achieved through a dedicated human coach who partners with the participant for the entire Pivot journey. Communication with one’s coach is conducted through asynchronous text messaging within the Pivot app, allowing the participant to initiate outreach or respond to their coach using a modality and timeline that fits into their life. To increase reach, Pivot has been designed to support users along the entire spectrum of readiness to quit, from being unsure or ambivalent to being highly motivated. Pivot does not assume participants are ready to quit smoking at entry and, consequently, does not start with choosing a quit date or developing a quit plan but rather begins with self-exploration and awareness building.

A feasibility study of the first stage of Pivot (ie, Explore) examined program engagement, changes in attitudes toward quitting, self-reported changes in smoking behavior, and program acceptability [22]. Engagement rates over the 9 days of Explore were as follows: ≥80% of participants (34 to 39 of 41) used the CO sensor ≥1 time per day and over 55% (23 to 27 of 41) used it ≥5 times per day; all 9 in-app activities had completion rates of ≥80% (33 to 40 of 41). Furthermore, coach-initiated contacts received response rates of ≥73% (30 to 39 of 41). Results also yielded significant positive changes in attitudes toward quitting smoking from baseline to study exit, including increased readiness to quit, lower perceived difficulty quitting, and greater expectations of success. At study exit, 78% of participants (32/41) reported that they had decreased the number of cigarettes smoked per day since beginning the study. They also rated program quality and satisfaction as very high. Although these results are promising, there were some limitations. First, this was an initial feasibility study that examined only the first stage of Pivot: Explore. Additional research is needed to examine longer-term engagement throughout the Pivot program. Finally, although participants reported decreasing cigarette smoking, this feasibility study did not include more robust indicators of changes in smoking such as number of quit attempts and 7- and 30-day PPA.

### Objectives

This pragmatic, exploratory study is the first evaluation of the Pivot program in the hands of its intended users. The purpose of this study was to assess participant engagement, changes in attitudes toward quitting, and changes in smoking behavior.

## Methods

### Study Design

This was a prospective open-label, single-arm study of Pivot (Carrot Inc). The study was conducted as a pragmatic, exploratory study to assess the initial performance of the Pivot program. The study examined engagement (ie, weeks active in the program and stage progression), changes in attitudes toward quitting smoking (ie, readiness to quit, confidence about quitting, and anticipated difficulty quitting), and changes in smoking behavior (ie, quit attempts, reduction in cigarettes smoked per day, and 7- and 30-day PPA).

### Consent and Ethical Approval

All participants provided electronic informed consent before participation. The study was reviewed and approved by the Solutions institutional review board (Little Rock, AR, USA) protocol number 2017/09/22 and registered with Clinicaltrials.gov NCT03295643.

### Eligibility and Recruitment

Participants were enrolled from October 2017 to March 2018. Potential participants were identified via advertisements on Web media (ie, Facebook, Instagram, Twitter, Google Ads, Reddit, and smokefree.gov), with a link to an online screening form. Contact information and data on age, sex, smartphone ownership, employment status, and smoking behavior were collected. To be eligible for participation, individuals had to meet all of the following eligibility criteria: aged 18 to 65 years, English speaking, smoke ≥5 cigarettes per day (CPD), own and use a compatible smartphone (iPhone 5 and above, operating system iOS 9.0 and above, or Android 4.4 and above, operating system Android 4.4 and above), be employed for ≥20 hours a week, and live in the United States. Although we aim for broad availability of Pivot through multiple channels such as private and public insurers, direct-to-consumer, and not-for-profit foundations, Pivot will initially be available to individuals through their employers (self-insured employers or employee wellness programs). As such, we applied the employment requirement to assess Pivot in individuals closely aligned with Pivot’s initial user population. This approach was informed by recent reports, which indicate that 75% of US employers offer wellness resources and/or a general wellness program with representation across all occupational and wage groups [23-25]. In addition, in most US states, being employed 20 hours per week or more is a requirement for benefits eligibility, including access to wellness offerings.

Nonproportional quota sampling was employed with percentage limits applied to age, CPD, stage of change, and sex (Table 1). The eligibility criteria and nonproportional quota sampling were used to achieve a study population that reflects the initial targeted commercialized population (ie, employed adult smokers likely to have access to a wellness benefit offering, representing the spectrum of readiness to quit). Individuals were called on a first-come-first-serve basis with nonproportional quota sampling enrollment guidelines applied. Initially, Pivot was released for iPhone only. Toward the end of enrollment, Pivot became available for use on the Android platform, and enrollment shifted accordingly.

Those who met the eligibility criteria and met the nonproportional quota sampling requirements were contacted by phone to describe the study and allow potential participants to ask questions. Those expressing interest in study participation were emailed a Web address where they could register and complete the electronic informed consent. Participants did not have to indicate intent to quit smoking as a condition of study participation.

### Onboarding

Upon providing electronic informed consent and completing the online registration, participants received an email with a link to the baseline questionnaire, which assessed demographics, smoking history, and attitudes toward quitting (ie, readiness to quit, confidence to quit, and perceived difficulty of quitting). They were also mailed the CO breath sensor and instructions to download the Pivot program onto their smartphone. Getting started in Pivot was a self-guided process. Participants used the provided instructions for downloading the Pivot program on their smartphone and pairing the CO sensor to the Pivot app. They also had access to customer service as needed. Once this process was complete, the participant was considered to have onboarded. After successful onboarding, they were paired with a live, dedicated coach who provided one-on-one support over the course of the study.

**Table 1:** Nonproportional quota sampling enrollment: target and actual values (n=319).

Category		Targeted enrollment values (%)	Actual enrollment values, n (%)
**Age (years)**			
	18-29	<20	24 (7.5)
	30-60	≥70	281 (88.1)
	≥61	<10	14 (4.4)
**Cigarettes per day**			
	5-10	<25	68 (21.3)^a^
	11-30	≥65	236 (74.0)
	>30	<10	15 (4.7)
**Stage of change**			
	Intend to quit within 30 days	≥20	107 (33.5)
	Intend to quit within 6 months	≥20	201 (63.0)
	Not thinking of quitting	<20	11 (3.5)
**Sex**			
	Female	40-60	184 (57.7)

^a^One participant reported smoking 5 cigarettes per day at screening, then entered 4 cigarettes per day on the baseline questionnaire.

**Figure 1:**
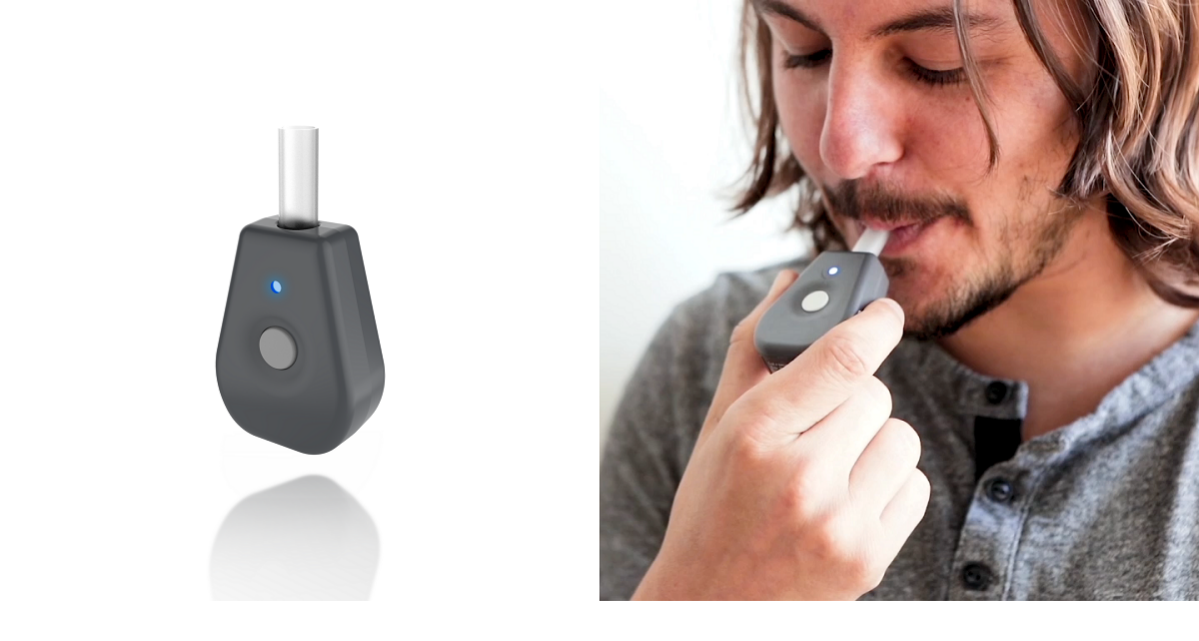
Pivot carbon monoxide breath sensor.

### Pivot Program

Pivot is a comprehensive digital solution that includes (1) the first FDA-cleared, over-the-counter CO breath sensor (Figure 1), which communicates with a smartphone and app via Bluetooth; (2) the multiphase Pivot mobile app; and (3) dedicated human coaching delivered one-on-one through in-app text messaging. The program has been developed for commercialization and designed for delivery in the context of employee wellness programs and health plans. Pivot leverages evidence-based principles and clinical best practices. Specifically, Pivot uses the USCPG-recommended 5 As (Ask, Advise, Assess, Assist, and Arrange), tailors on readiness to quit [4], encourages the use of FDA-approved pharmacotherapy [4,26-28], capitalizes on effective methods for smoking cessation (eg, motivational interviewing, cognitive behavioral therapy, and self-determination theory) [4,29-31], and provides behavioral counseling through a live, dedicated coach [4,28,32,33].

**Figure 2:**
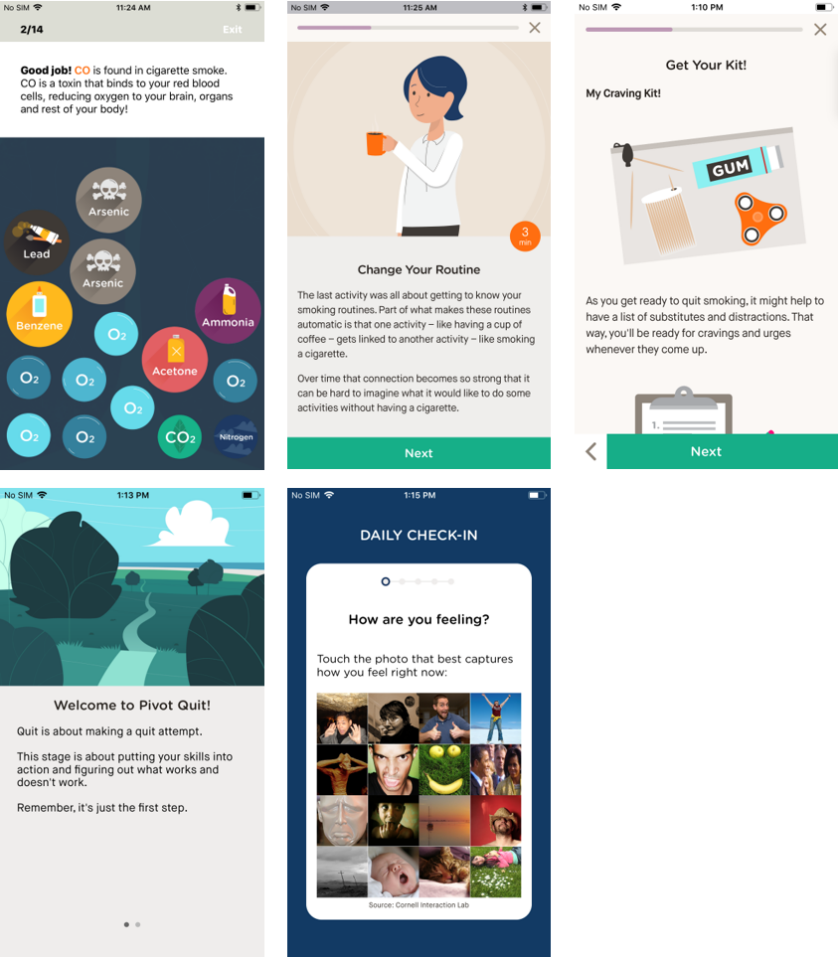
Sample screenshots from the Pivot app, different stages.

The Pivot journey consists of 5 sequential stages (Explore, Build, Mobilize, Quit, and Secure). To advance to the next Pivot stage, the preceding stage must be completed. Pivot begins with *Explore* (9 days), which is designed for anyone who smokes, to raise awareness and interest in moving forward. In Explore, users take samples with the Pivot Breath Sensor, log cigarettes, get to know their coach, and complete daily activities to understand their smoking patterns and explore how smoking affects their lives. The second phase of Pivot is *Build* (1 day to 4 weeks), which is tailored to users’ readiness, motivation, and confidence. Build culminates with users setting a quit date and building a quit plan. Next is *Mobilize* (7 days), which provides opportunities for users to put into practice individual elements of their quit plan, one at a time, in preparation for quit day. The fourth phase of Pivot is *Quit* (7 days), which begins on the user’s selected quit day and continues through the first week of living smoke-free. Quit incorporates a daily check-in feature to allow users to track their progress and set daily goals to reinforce the idea of quitting as a process. *Secure* (11 weeks) is a natural extension of Quit and focuses on supporting users in developing internal, sustainable motivation to stay smoke-free for good. With continued coaching support, self-monitoring, and practice, Secure is designed to help Pivot’s newly smoke-free users learn to navigate the challenges that come in the first few months after quitting. Sample screenshots from the Pivot app from different stages are shown in Figure 2.

Throughout the Pivot program, participants could provide breath samples, log cigarettes, complete in-app activities, and interact with their coach via in-app chat. During the study, the suggested breath sampling frequency was once per hour while awake during Explore through Mobilize, decreasing to a few breath samples per day thereafter. This was discussed during the screening call with acknowledgment that breath sampling should ultimately be undertaken in a way that works with each participant’s schedule. Participants were also encouraged to log all cigarettes they smoked using the in-app logging feature, preferably as soon as possible after smoking. A daily push notification alerted participants to their daily in-app activity (Explore through Quit) or check-in (Quit and Secure). Finally, participants received behavioral counseling via coaching throughout the entire program. Coach-initiated contact included outreach 3 times a week from Explore through the first 30 days in Secure, once per week for the next 30 days in Secure, and every other week for the last 30 days in Secure. Participants could initiate contact with their coach as frequently as desired. Coaching was undertaken through asynchronous in-app text messaging, thus allowing participants to respond to coach-initiated contact or to initiate contact with their coach whenever it was convenient for them.

### Procedure

The Pivot program was designed to be 14.5 to 18.5 weeks in duration, depending on participant navigation of the program stages (Build lasts 1 to 28 days depending on participant readiness and desired pace). Participants were asked to complete electronic questionnaires in-app and online through Survey Monkey at baseline, upon completion of each stage, and at the end of Pivot. Participants were notified of the online questionnaires via email with a link to the questionnaire. Automated email reminders were sent daily until online questionnaire completion, the emailing of the next study questionnaire, or in the case of the final questionnaire, until the end of the study period. Participants were compensated US $10 to US $50 per completed study questionnaire and US $50 for returning the CO breath sensor for up to a total of US $265, using Visa gift cards. Compensation was not associated with use of the various components of Pivot, level of engagement, or smoking/quitting status.

### Data Collection

Data were collected electronically through participant input in the Pivot online registration form, Pivot app, and online questionnaires. Study data were imported directly into a secure database (PostgreSQL, PostgreSQL Global Development Group).

### Outcome Variables and Measurement

#### Baseline Characteristics

Baseline characteristics included demographic information (age, sex, race or ethnicity, household income, and education) and smoking behavior data (cigarettes smoked per day, duration of smoking, and number of quit attempts over the past 12 months).

#### Engagement

A total of 3 metrics of engagement were assessed: Pivot stage completion, weeks of active engagement, and program completion. Pivot stage completion is defined as the percent of participants who completed each stage based on the number of those who onboarded. Weeks of active engagement was defined as doing at least 1 of the following: doing a breath sample, logging a cigarette, starting or completing a daily activity, challenge or check-in, or messaging one’s coach. Opening the app without doing one of the aforementioned actions and receipt of a message from one’s coach did not count as engagement. Finally, program completion was defined as those who completed onboarding and completed Explore through Secure. Completing Secure was defined as (1) having been in Secure for 11 weeks, (2) having engaged with the program at least once during the 11 weeks of Secure as defined above, and (3) providing smoking status (CPD and 7- and 30-day point prevalence) in the final questionnaire.

#### Attitudes Toward Quitting

Measurements assessing attitudes toward quitting included readiness to quit/stage of change [34,35], confidence to quit, and anticipated difficulty maintaining quit status [36,37]. These measures were chosen to reflect a range of psychosocial indicators that have been shown to influence the likelihood of attempting to quit smoking and/or cessation (ie, motivation and self-efficacy). Participants completed the readiness to quit/stage of change item at baseline and the end of Explore. To complete the second phase of Pivot, Build, participants had to have selected a quit date within the next 14 days. Therefore, it did not make sense to ask participants the readiness to quit/stage of change item beyond the end-of-Explore survey. Confidence to quit and anticipated difficulty maintaining quit status were assessed at baseline and at the end of Explore, Build, and Mobilize. The specific items used to assess participant attitudes are detailed in Table 2.

**Table 2:** Measurements assessing attitudes toward quitting.

Question	Answer options/scale
Are you seriously thinking of quitting smoking? (stage of change)	Yes, within the next 30 days
	Yes, within the next 6 months
	No, not thinking of quitting
If you were to quit smoking right now, how successful would you be?	Scale 1 to 10 (1=not at all successful, 10=completely successful)
If you were to quit smoking right now, how difficult do you think it would be to stay smoke-free?	Scale 1 to 10 (1=really hard to stay quit, 10=really easy to stay quit)

#### Changes in Smoking Behavior

A total of 4 metrics were used to assess changes in smoking behavior: smoking reduction, quit attempts, and 7-day and 30-day PPA. At the end of each stage, monthly in Secure, and on the final questionnaire, participants were asked the number of cigarettes smoked per day. *Smoking reduction* was evaluated in all participants before Quit and in the subset of participants who never achieved 7-day PPA using the following metrics: change in CPD, percentage change in CPD, and percentage of participants who achieved ≥50% reduction in CPD. *Quit attempt* was defined as going at least 1 day without smoking cigarettes, even a single puff. *Point-prevalence abstinence (7-day and 30-day)* was assessed as a primary outcome on the final study questionnaire. We also asked PPA for secondary outcomes at the end of Quit (7-day PPA) and monthly in Secure. Participants were considered to have achieved 7-day (30-day) PPA if they answered “no” to the following question: “In the last 7 (30) days have you smoked any cigarettes, even a single puff?” As the Pivot program has no face-to-face contact, and data collection is achieved through remote means using the app and electronic questionnaires, biochemical verification of smoking status was not pursued in accordance with previous recommendations [38].

#### Sample Size

Previous evaluation has shown that changes in attitudes toward quitting are meaningful predictors of quit attempts [39]. On the basis of a previous assessment of 41 individuals using the first stage of Pivot (Explore), we estimated that the mean (SD) change in ratings assessing attitudes toward quitting (confidence to quit and expected difficulty maintaining quit) would be ≥1 (4) just before reaching Quit [22]. On the basis of these estimates, there was 80% power to detect a significant difference in these ratings with a sample size of 101. As this was an initial study of the complete Pivot program, and in the context of known high attrition rates with mobile health apps [40,41], we applied conservative retention estimates drawn from other similar studies. Specifically, the target enrollment of 310 was estimated to yield at least 100 participants still engaged at the end of Secure.

### Statistical Analyses

Statistical analyses were conducted using all available data. For engagement, data were collected through the Pivot app to capture stage completion, weeks of active engagement, and program completion. Changes in attitudes toward quitting were assessed from baseline to the end of Explore, Build, and Mobilize (ie, pre-Quit); changes in cigarettes smoked per day were assessed from baseline to the end of each Pivot stage and the final questionnaire. Participants served as their own controls, and comparisons were made to no change. To evaluate changes in attitudes or cigarettes smoked per day over time, repeated measures linear mixed model analyses were performed using a compound symmetric correlation matrix to model the repeated measures within subjects. As these measurements were taken at the same Pivot stage (not necessarily the same time), stage was used as a surrogate for time. To make specific comparisons across time, *F* statistics were computed using the results from the model. To determine whether the effects over time were the same among subgroups, subgroup and time by subgroup interaction terms were added to the models with time. Analyses were conducted to calculate mean (SD) for normally distributed variables for actual data or mean (SE) for modeled data and median (interquartile range) values in instances of non-normally distributed variables. Furthermore, one-sample *t* tests were used for numerical data. Fisher exact or chi-square tests were used for categorical data. McNemar test was used for 2-category match-paired data. Cohen kappa statistic was used for 3-category match-paired data. Analyses were conducted using SAS Version 9.4 (SAS Institute). Statistical significance was set at *P*<.05.

In the assessment of cessation (PPA), 2 sets of analyses were performed. In the first, an ITT analysis, individuals who did not respond to PPA questions were assumed to be smoking. The ITT analysis was performed as a standard assessment with long-standing use in traditional clinical studies. However, this method is subject to biases in effect size estimates and may lead to errors [42,43]. Consequently, a study completer analysis was also performed, which only included individuals who completed the final questionnaire. Participants were sent the final questionnaire regardless of whether or not they completed the Pivot program. For additional assessments performed at the end of the study (quit attempts, CPD, and smoking reduction), a study completer analysis was performed. This analysis approach comports with previous reports assessing app-based digital cessation programs [14,15].

## Results

### Enrollment

**Figure 3:**
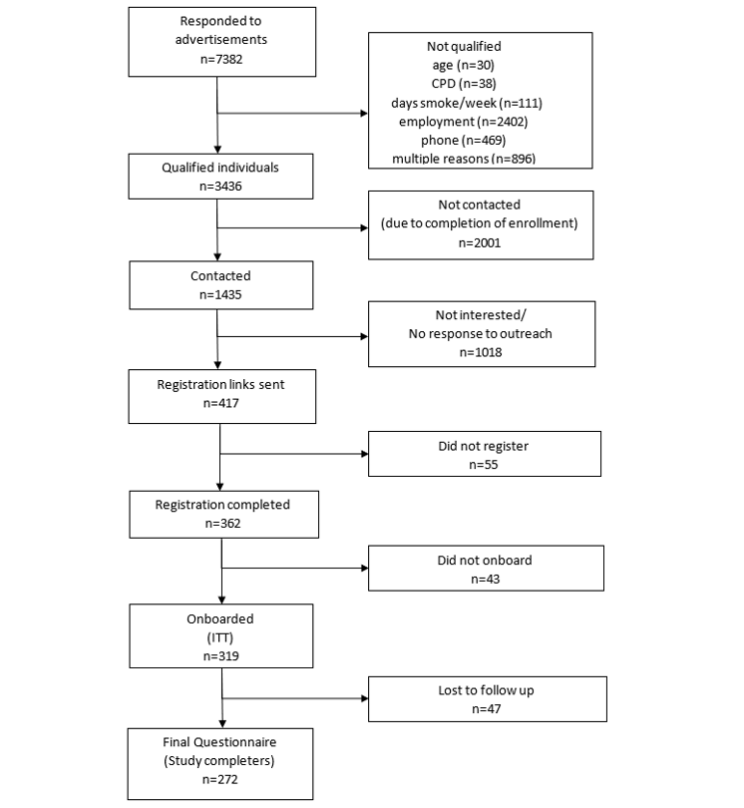
Consolidated Standards of Reporting Trials flow diagram of participants.

From October 2017 to March 2018, 7382 online screening forms were received; 3436 met the screening eligibility criteria, and 1435 received outbound phone calls from study staff. Most phone calls were not answered or returned. After the phone calls, registration links were emailed to 417 potential participants; 362 completed the online registration and informed consent. A total of 319 participants completed onboarding and comprised the ITT sample. Moreover, 85.3% (272/319) of the participants completed the final questionnaire and comprised the study completer sample. A total of 47 participants were lost to follow-up or withdrew consent. Study enrollment and attrition are depicted in the participant flow diagram in Figure 3.

### Baseline Characteristics

The study sample consisted of 57.7% women (184/319), had a mean age of 42.8 (SD 10.2) years, smoked a mean of 17.7 (SD 7.6) CPD at baseline, and had been smoking for an average of 26.4 (SD 10.7) years. Most study participants (294/319, 92.2%) used iPhones.

At baseline, one-third of participants (107/319, 33.5%) indicated they were seriously thinking of quitting smoking in the next 30 days. Two-thirds of participants (212/319, 66.5%) did not intend to quit smoking within the next 30 days. Specifically, 63.0% (201/319) indicated they were thinking of quitting in the next 6 months, and 3.5% (11/319) indicated they were not seriously thinking of quitting smoking. On average, participants had made 2.1 (SD 3.3) quit attempts over the past 12 months. Study demographic details are provided in Table 3.

**Table 3:** Baseline demographics.

Demographics		Statistics
Completed onboarding, n		319
Female, n (%)		184 (57.7)
**Age (years), mean (SD)**		42.8 (10.2)
	20-29, n (%)	24 (7.5)
	30-39, n (%)	108 (33.9)
	40-49, n (%)	103 (32.3)
	50-59, n (%)	63 (19.7)
	60-69, n (%)	21 (6.6)
Cigarettes smoked per day, mean (SD)		17.7 (7.6)
Years smoking, mean (SD)		26.4 (10.7)
Quit attempts in last 12 months, mean (SD)		2.1 (3.3)
**Smartphone, n (%)**		
	iPhone	294 (92.2)
	Android	25 (7.8)
**Ethnicity, n (%)**		
	White	264 (82.8)
	African American	22 (6.9)
	Hispanic	15 (4.7)
	Asian	5 (1.6)
	American Indian or Alaska Native	4 (1.3)
	Native Hawaiian or other Pacific Islander	2 (0.6)
	Other	7 (2.2)
**US region, n (%)**		
	South	123 (38.6)
	Midwest	73 (22.9)
	West	68 (21.3)
	Northeast	55 (17.2)
**Highest level of education or degree attained, n (%)**		
	Professional or doctorate degree	8 (2.5)
	Master’s degree	18 (5.6)
	Bachelor’s (4-year) degree	70 (21.9)
	Associate’s (2-year) degree	52 (16.3)
	Some college	112 (35.1)
	High school/GED^a^	56 (17.6)
	Some high school	3 (0.9)
**Annual household income (US dollars), n (%)**		
	Less than $25,000	30 (9.4)
	$25,000-$34,999	48 (15.0)
	$35,000-$49,999	58 (18.2)
	$50,000-$74,999	60 (18.8)
	$75,000-$99,999	56 (17.6)
	$100,000-$149,999	40 (12.5)
	$150,000 or more	17 (5.3)
	Prefer not to answer	10 (3.1)
**Are you seriously thinking of quitting smoking? n (%)**		
	Yes, within the next 30 days	107 (33.5)
	Yes, within the next 6 months	201 (63.0)
	No, not thinking of quitting	11 (3.5)
**Attitude toward smoking ratings, mean (SD)**		
	If you were to quit smoking now how successful would you be? (1=not successful at all, 10=completely successful)	4.2 (2.7)
	If you were to quit smoking right now how difficult do you think it would be to stay smoke-free? (1=really hard; 10=really easy)	3.1 (2.5)

^a^GED: General Education Diploma.

### Engagement

**Figure 4:**
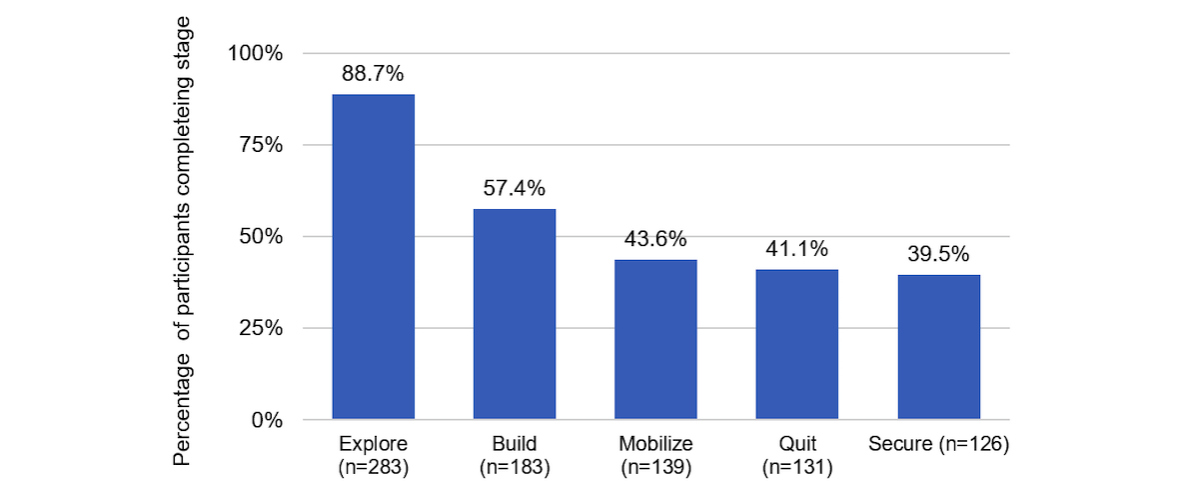
Proportion of participants who completed each Pivot stage, by those who onboarded (n=319).

The proportion of participants who completed each Pivot stage is detailed in Figure 4. As shown, after some drop-off in Build, retention rates remained relatively stable for the remainder of program participation. Overall, 39.5% (126/319) of participants who onboarded completed the Pivot program.

Participants were active in the program for a mean of 12.4 (SD 7.1) weeks. Time to complete each stage varied for some from original projections with participants taking longer in Mobilize, Quit, and Secure (Table 4).

**Figure 5:**
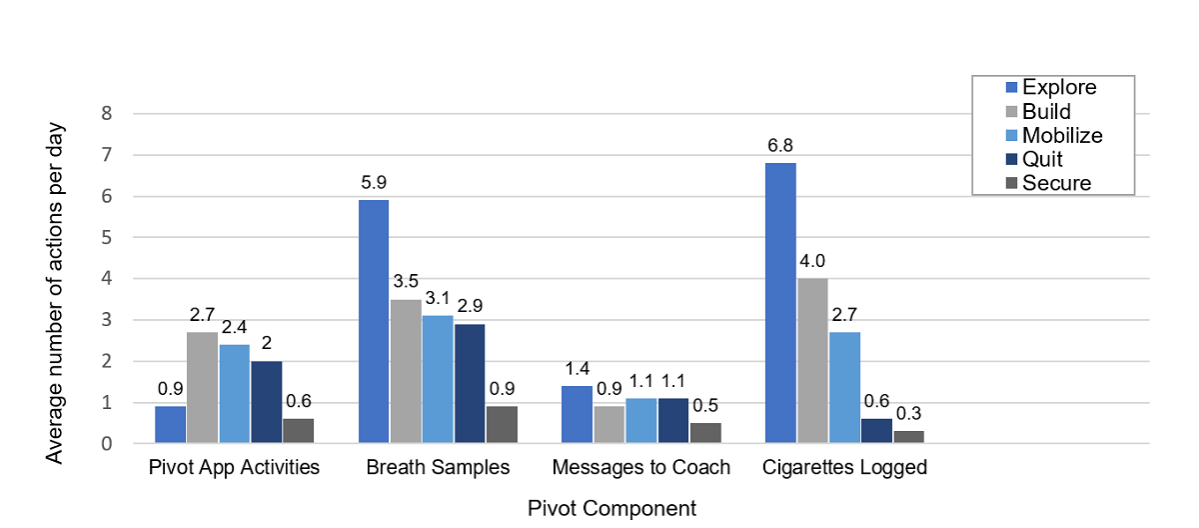
Participant engagement with the different components of Pivot; average actions per day, by stage, for all participants who entered stage.

Beyond active engagement by week, as a secondary analysis, we explored patterns of daily engagement to gain a more detailed understanding of how participants interacted with Pivot (Figure 5). Overall, participants consistently used all Pivot components throughout their engagement with the Pivot program. Notable use pattern variation included performance of the highest average number of daily breath samples in Explore, completion of the highest average number of daily Pivot app activities in Build, and an overall decrease in daily logging of cigarettes from one stage to the next. These use patterns are expected. Specifically, breath sampling is particularly salient to Explore’s focus on self-awareness and exploration of how smoking affects one’s life. Build is a time for learning and skill building, which are primarily accomplished through completion of daily app activities. Cigarette logging would decrease both as a result of actual decreases in smoking and decreased compliance with logging during advancement in the program. In addition, there was continued but decreased use of all program components in Secure, consistent with an expected decrease in need for program intensity as participants achieve cessation.

**Table 4:** Projected and actual days spent in each Pivot stage.

Stage	Projected days	Actual days, mean (SD)
Explore	9	9.3 (4.1)
Build	1-28	19.8 (26.2)
Mobilize	7	18.0 (23.0)
Quit	7	9.1 (10.0)
Secure	77	87.3 (7.7)

### Change in Attitudes Toward Quitting

Change in readiness to quit (stage of change) was assessed at baseline and at the end of Explore (Table 5). Readiness to quit improved with 55.9% (157/281) of respondents indicating they were thinking of quitting in the next 30 days at the end of Explore compared with 33.5% (94/281) at baseline (*P*<.001). At the end of Explore, 28.1% (79/281) increased their readiness to quit, 66.2% (186/281) stayed the same, and 5.7% (16/281) decreased (*P*<.001).

**Figure 6:**
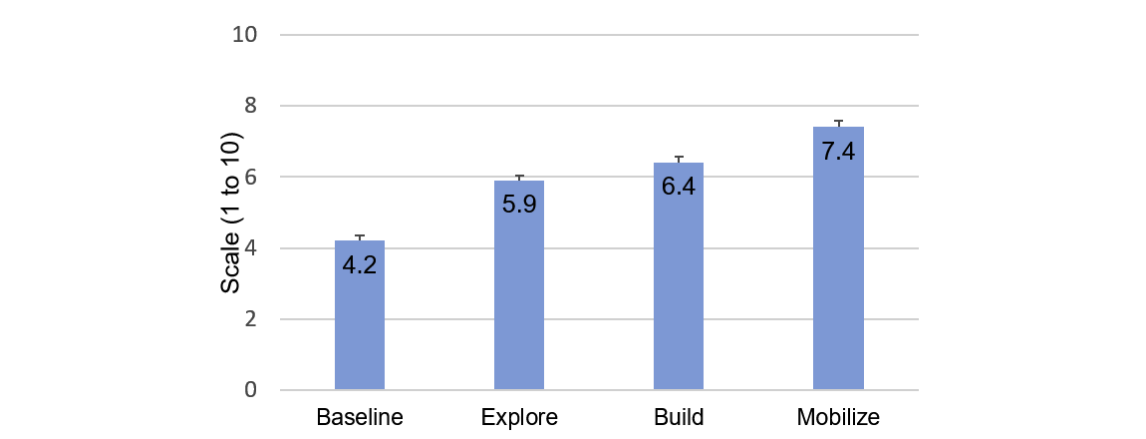
Changes in confidence to quit. Estimate of means and standard errors based on linear mixed model.

**Figure 7:**
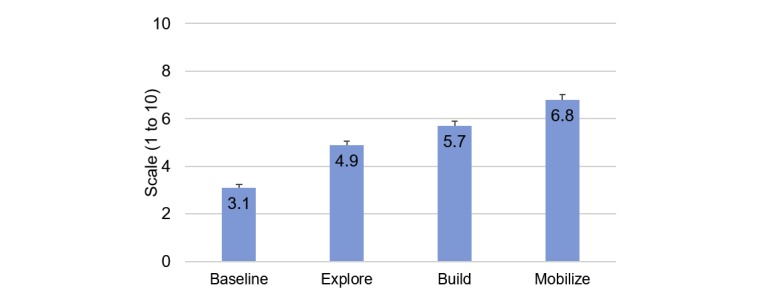
Changes in perceived difficulty of staying quit. Estimate of means and standard errors based on linear mixed model.

Participants’ confidence to quit and perceived difficulty of quitting also improved (Figures 6 and 7). In repeated measures linear mixed model analyses, end-of-stage results were compared with baseline and each other; all changes were statistically significant (*P*<.001, except for end of Build vs end of Explore, where *P*=0.01 for confidence to quit).

**Table 5:** Change in readiness to quit from baseline to the end of Explore.

Readiness to Quit: Baseline		Readiness to Quit^a^: End of Explore, n (%)			
		Yes, within the next 30 days	Yes, within the next 6 months	No, not thinking of quitting	Total
Yes, within the next 30 days		82 (29.2)	12 (4.3)	0 (0)	94 (33.5)
Yes, within the next 6 months		74 (26.3)	98 (34.9)	4 (1.4)	176 (62.6)
No, not thinking of quitting		1 (0.4)	4 (1.4)	6 (2.1)	11 (3.9)
Total		157 (55.9)	114 (40.6)	10 (3.6)	281 (100.0)^b^

^a^Readiness to Quit assessed via stage of change question: Are you seriously thinking of quitting smoking? A. Yes, within the next 30 days; B. Yes, within the next 6 months; C. No, not thinking of quitting.

^b^283 participants completed Explore; however, 2 of these participants did not answer this question on the end of Explore questionnaire.

### Changes in Smoking Behaviors

#### Change in Cigarettes Per Day by Stage

Repeated measures linear mixed model analysis was performed, with projected end-of-stage CPD values compared with baseline and each other (Table 6). End-of-stage percent changes in CPD were statistically significant (all *P*<.001 with *P*=.003 for Mobilize vs Build), except at the end of Explore versus baseline in those ready to quit in the next 30 days (percentage change in CPD=−10.2%, *P*=.13). Among those who completed Mobilize and provided data (n=139), CPD were reduced by 50.7% (SD 32.6) from baseline to immediately before entering Quit.

Using repeated measures linear mixed model analysis, 31.8% of study participants were projected to reduce their CPD by ≥50% before entering Quit (Table 7). Among those who completed Mobilize and provided data, 53.2% (74/139) reduced their CPD by ≥50% before entering Quit. At the end of the study, among the study completers who did not achieve at least 7-day PPA (n=170), CPD were reduced by 29.1% (SD 34.5), and 25.9% (44/170) reduced their CPD by ≥50%. Pre-Quit (Explore, Build, and Mobilize) there were no statistically significant differences in CPD reduction rates or in the proportion of participants reducing CPD by ≥50% between those ready and not ready to quit in the next 30 days at entry.

**Table 6:** Average percentage change in cigarettes per day (CPD) by pre-Quit stage, repeated measures linear mixed model analysis.

Group	Pivot stage		
	Explore	Build	Mobilize
All, mean % (SE)	−12.6 (2.5)	−30.7 (2.9)	−44.5 (3.0)
Baseline Readiness to Quit: within the next 30 days, mean % (SE)	−10.2 (6.6)	−27.7 (7.1)	−45.1 (7.4)
Baseline Readiness to Quit: *not* within the next 30 days, mean % (SE)	−13.8 (2.0)	−32.1 (2.4)	−44.0 (2.5)

**Table 7:** Proportion of participants who reduced cigarettes per day (CPD) by ≥50% by pre-Quit stage, repeated measures linear mixed model analysis.

Group	Pivot stage		
	Explore	Build	Mobilize
All	11.3 (8.1-15.6)	21.2 (16.8-26.4)	31.8 (26.6 -37.5)
Baseline Readiness to Quit: within the next 30 days, estimated percentage (95% CI)	14.9 (9.0-23.6)	22.3 (15.0-31.9)	37.2 (28.1-47.4)
Baseline Readiness to Quit: *not* within the next 30 days, estimated percentage (95% CI)	9.5 (6.1-14.6)	20.6 (15.5-27.0)	29.1 (23.1-36.0)

**Table 8:** Among participants with increased motivation to stop smoking in Pivot, what was the top reason?

Reason	Statistics, n (%)
Using the sensor to measure carbon monoxide in my breath	80 (34.3)
Trying a variety of strategies to reduce or quit	61 (26.2)
Learning about the effects of smoking through daily activities	38 (16.3)
Coaching	33 (14.2)
Other	16 (6.9)
Receiving support from friends, family, and others	5 (2.1)
Total	233 (100)

#### Quit Attempts

Overall, 79.4% (216/272, study completers) reported making at least 1 quit attempt during their participation in the Pivot program. On average, study completers made 2.4 (SD 3.1) quit attempts during Pivot. Among the participants who, at baseline, were planning to quit smoking within the next 30 days, 89.1% (82/92, study completers) made a quit attempt during the study. Among the participants who, at baseline, were *not* planning to quit smoking within the next 30 days, 74.4% (134/180, study completers) made a quit attempt during the study.

#### Point Prevalence Abstinence (7-Day and 30-Day)

At the end of the study, 32.0% (102/319) achieved 7-day PPA and 27.6% (88/319) achieved 30-day PPA using an ITT analysis. Using data from study completers, 37.5% (102/272) achieved 7-day PPA and 32.4% (88/272) achieved 30-day PPA.

Subset analyses of those who were ready to quit within the next 30 days at baseline versus those who were not ready to quit demonstrated similar cessation rates between the 2 groups. Using an ITT analysis, among those who, at baseline, were ready to quit smoking in the next 30 days, 32.7% (35/107) achieved 7-day PPA and 27.1% (29/107) achieved 30-day PPA; for those, at baseline, who were *not* ready to quit smoking in the next 30 days, end-of-study 7-day and 30-day PPA were 31.6% (67/212) and 27.8% (59/212), respectively. A similar pattern emerged for the study completer analysis. Among those who, at baseline, were ready to quit smoking in the next 30 days, 38.0% (35/92) achieved 7-day PPA and 31.5% (29/92) achieved 30-day PPA; for those, at baseline, who were not ready to quit smoking in the next 30 days, end-of-study 7-day and 30-day PPA were 37.2% (67/180) and 32.8% (59/180), respectively. None of these differences in PPA between subsets were statistically significant.

In addition to the primary endpoint with the end-of-study questionnaire, 7-day PPA and 30-day PPA data were also collected as secondary outcomes periodically throughout the Pivot program: at the end of Quit (7-day PPA only) and in Secure at 30, 60, and 90 days (7-day and 30-day PPA). Using all available data, 36.1% (115/319) achieved 7-day PPA and 31.0% (99/319) achieved 30-day PPA at some point during the study.

### Participant Feedback

Participants were asked to consider the various components of Pivot. Among the participants who reported Pivot increased their motivation to stop smoking (85.7%, 233/272), using the breath sensor was the most common reason for the increased motivation (Table 8). Trying a variety of strategies to reduce or quit and learning about the effects of smoking through daily activities were the second and third most common reasons, respectively.

## Discussion

### Principal Findings

This was the initial study of the Pivot program; a comprehensive, multiphase digital smoking cessation program that includes a mobile CO breath sensor, the Pivot app, and dedicated human coaching. The majority (212/319, 66.5%) of participants were not ready to quit in the next 30 days at study entry. Overall, 39.5% (126/319) of participants completed the Pivot program. Participants were active in the program for an average of 12.4 weeks.

There was a positive, statistically significant shift in attitudes toward quitting, including readiness to quit/stage of change, confidence in quitting, and perceived difficulty of staying quit. With regard to changes in smoking behavior, 53.2% of participants who completed Mobilize decreased CPD by at least half just before entering Quit. Moreover, 30-day PPA was achieved by 27.6% (88/319) and 32.4% (88/272) of participants for ITT and study completer analyses, respectively. Notably, 30-day abstinence rates were comparable among those who were and were *not* ready to quit in the next 30 days at baseline: 27.1% (29/107) versus 27.8% (59/212; ITT), respectively. Among the study completers who did not achieve abstinence, 25.9% (44/170) reduced their CPD by ≥50% by the end of the study.

### Readiness to Quit

This study was unique in its inclusion of individuals who were not ready to quit smoking in the next 30 days. This study design element reflected an overarching goal of Pivot to engage individuals along the entire spectrum of readiness to quit. Prochaska et al estimated that approximately 20% of smokers are thinking of quitting smoking in the next 30 days, 35% to 40% are thinking of quitting in the next 6 months, and 40% to 45% are not seriously thinking of quitting. They noted that cessation professionals, “approaching patients and settings only with action-oriented programs are likely to under-serve or misserve the majority of their target population” [44]. Despite the fact that the USCPG provides clear guidance on how to work with those who are ambivalent or otherwise not ready to quit in the next 30 days [4], few programs actively recruit or even include these individuals. Pivot had high enrollment of individuals not ready to quit in the next 30 days and comparable program retention and cessation rates between these individuals and those who were ready to quit in the next 30 days. This suggests Pivot addresses this historical shortcoming of focusing solely on those smokers who are ready to quit in the short term and has the potential to increase reach by effective inclusion of a larger proportion of the smoking population.

### Comparison With Prior Work

In addition to inclusion of individuals who were not ready to quit smoking in the next 30 days, differentiating aspects of Pivot include the mobile CO breath sensor and dedicated in-app human coaching. These differences, along with those in program duration and study population characteristics such as employment status, age, and baseline CPD, limit comparability of these programs. Acknowledging these differences, Table 9 is included to provide context for current and previous abstinence rates from app-based cessation programs. Overall, Pivot abstinence rates are favorable when assessed among app-based cessation programs.

Recently, Krishnan et al assessed the use of a personal CO breath sensor (iCO monitor, Bedfont Scientific Ltd) with the COach2Quit app. Participants were randomized to receive brief advice (control, n=52) or brief advice plus the iCO monitor with the COach2Quit app (intervention, n=50). Participants in the intervention group were sent reminders from the app to use the breath sensor twice per day. On the basis of the user’s CO result after a breath sample, the app sent messages from a predefined text library and provided a graphical display of the CO readings. At 30-day follow-up, 1 participant in each study arm had quit smoking. There were no significant differences between study groups in changes in CO levels or CPD between baseline and 30-day follow-up [45]. The use of a personal CO breath sensor was incorporated in this study and our study. However, there were several notable differences in the programs and study populations, including the intensity of and delivery mechanism of counseling, suggested breath sampling frequency, the app-delivered Pivot journey, and study population demographics (age, employment, and education levels). These differences may have contributed to the different outcomes.

### Adherence and Attrition

Poor adherence and attrition are known problems with technology-based treatment platforms such as apps. Ubhi et al evaluated SmokeFree28, a 28-day app-based cessation program. Among the 1170 participants, 470 (40.2%) used the app for 7 days or more and only 226 (19.3%) used it for 28 days or more [40]. Iacoviello et al reported on the use of an app-based cessation program by 416 smokers. In that program, users chose a quit date 7 to 21 days from enrollment with study endpoint data collected at 8 weeks. Study participants used the program for an average of 5.3 weeks [15]. In Pivot, participants were actively engaged with the program for an average of 12.4 weeks, indicating durable use among participants.

Of 99 participants in a study assessing a digital cessation program based on ACT, Bricker et al reported that at 2-month follow-up, 24% of participants had completed the program [14]. In this study, 39.5% (126/319) of participants completed the Pivot program. The greatest attrition occurred during the Build stage; 64.7% (183/283) of those who started Build completed it, in contrast to ≥76.0% for the other Pivot stages. To complete Build and advance in Pivot, one must choose a quit date and create a quit plan. This requirement reflects the overall linear program flow, which requires completion of one stage before moving on to the next. This design may deter further advancement in those not ready to quit, and it may also hinder engagement and data collection from individuals on the other end of the spectrum, those who make a quit attempt before reaching Pivot’s Quit phase. Although the overall Pivot program completion rate is favorable in the context of published rates, learnings from this study suggest the potential for app flow modifications that allow more flexibility in navigation, such that early quitters can access Quit content and those not ready to quit can maintain a sense of progress by accessing additional educational and motivational materials.

**Table 9:** 30-day point prevalence abstinence (PPA) in app-based cessation programs.

Author	Program components	Study characteristics	n	ITT^a^ 30-day PPA^b^, n (%)	Study completer 30-day PPA, n (%)
Bricker JB et al [14]	App: applies Acceptance and Commitment Therapy; users create a quit plan, complete 8 core modules, use “Urge Pass” tracker and “Anytime Coaching” (other ACT^c^-based activities)	Single arm pilot trial (assessment of program updates following previous RCT^d^); Mean age: 38 years; Female: 78%; Employed: 70%; CPD^e^: not reported, 32% smoked ≥1 pack per day; Seriously thinking of quitting in the next 30 days: 100%; Follow-up: 2 months	99	9 (9.1)	9 (11.0)
Iacoviello BM et al [15]	App: tailored quit plan of missions and personalized messages that adhere to USCPG^f^. Includes controlled breathing, personalized messaging, social engagement, encouragement of pharmacotherapy and medication adherence, and digital diversions	Single arm trial; Mean age: 36 years; Female: 59%; Employment not reported; CPD: 16.7; Seriously thinking of quitting in the next 30 days: 100%; Follow-up: 2 months	416	109 (26.2)	109 (29.9)
Marler JD et al (this study)	CO^g^ breath sensor—user tracks effect of smoking on breath CO; App: applies USCPG; includes daily activities, challenges, cigarette logging, encouragement of pharmacotherapy, quit plan, practice quits, and check-ins; Coaching: asynchronous, dedicated human coaching via in-app text messaging, based on Cognitive Behavioral Therapy and Self-Determination Theory	Single arm trial; Mean age: 43 years; Female: 58%; Employed: 100% (≥20 hours a week); CPD: 17.7; Seriously thinking of quitting in the next 30 days: 33.5%; Follow-up: 14.5 to 18.5 weeks	319	88 (27.6)	88 (32.4)

^a^ITT: intention-to-treat.

^b^PPA: point prevalence abstinence.

^c^ACT: Acceptance and Commitment Therapy.

^d^RCT: randomized controlled trial.

^e^CPD: cigarettes per day.

^f^USCPG: United States Clinical Practice Guideline.

^g^CO: carbon monoxide.

### Participant Experience: Carbon Monoxide Breath Sensing

Most study participants (233/272, 85.7%) indicated their motivation to quit smoking increased while using Pivot, reporting use of the breath sensor as the most common reason. Breath sensor use behavior during the study supports this preference and generally aligns with previous findings. Participants gave an average of 5.9 breath samples per day during Explore compared with 5.9 to 8.1 samples per day during Explore in a previous study [22]; comparison beyond Explore was not possible due to the shorter duration of the previous study. Assessment by Krishnan et al of a smoking cessation program that also utilized a personal CO breath sensor and accompanying app reported similar findings regarding reception of the technology; 91% of participants liked having the breath sensor and app to help them quit smoking, and 86% reported that using both motivated them to quit smoking [45]. Although the use of a personalized CO breath sensor for biometric feedback is a relatively new approach in smoking cessation programs, the data of this study are encouraging and engender additional questions for future study regarding the optimal use of this tool.

### Indicators of Future Outcomes of Behavior

Abstinence rates are paramount in smoking cessation research, and the abstinence rates in this study were favorable in the context of similar programs. Nonetheless, nicotine addiction is a disease state that rarely, if ever, exceeds a 50% cure rate per treatment attempt with smokers making an average of 6 to 29 quit attempts before quitting successfully [46]. Accordingly, indicators of future outcomes or behavior are of particular interest. In this study, 28.1% (79/281) of participants progressed from one stage of readiness to the next at the end of Explore; this stage advancement is important because it translates to a doubling of one’s chance of taking action in the next 6 months [44]. Among the study completers who did not achieve abstinence during the study, the average reduction in CPD at the end of the study was 29.1%, and 25.9% participants decreased their CPD by ≥50%. This behavior change is meaningful as the rates of quit attempts or cessation itself significantly increase among those who reduce CPD by ≥50% [47].

### Limitations

This study has a few limitations. First, the majority of participants (294/319, 92.2%) were iOS users. Ubhi et al assessed 1386 users of the SmokeFree28 app and reported differences in iOS and Android users. iOS users were more likely to have made a quit attempt within the last 12 months and set their quit date on the day of registration and were less likely to have used cessation medication to support their quit attempt compared with Android users [48]. Further study of Pivot in Android users will inform the understanding of if and how their outcomes differ from iOS users. Second, the eligibility criterion of employment ≥20 hours per week may limit the applicability of the results among the general public, particularly in individuals with lower socioeconomic status or serious mental health issues. Notably, despite the ≥20 hours per week employment eligibility requirement, approximately 25% of study participants had an annual household income less than US $35,000. As we did not collect information on mental health, we are unable to comment on the representation of individuals with mental health conditions in the study population. In addition, although we used the eligibility criterion of employment ≥20 hours a week as a proxy for individuals more likely to have access to an employee wellness program or employer-provided health care, study participants were not directly recruited from such employer-provided programs, thereby limiting direct conclusions in these populations. Third, although most study participants were not ready to quit in the next 30 days, only 3.5% (11/319) of all study participants fell into the specific category of “not thinking of quitting,” limiting assessment in this group. Overall, the study results should be considered in the context of the aforementioned participant smartphone, employment, and readiness to quit characteristics, with additional study of Pivot needed in a broader population of smokers.

In addition, participants were compensated for their participation. Although efforts were made to minimize the influence of payments, such as keeping individual payments under US $50, incorporating a several week delay between questionnaire completion and payment receipt, and not linking payment to use of program components or smoking outcomes, we cannot exclude some influence of study payment on participant behavior. Finally, the study was conducted in a pragmatic fashion to assess the Pivot program as it is used in real-world contexts. Pivot is characterized by a sequential multistage design. These stages were created with certain time frame estimates in mind, specifically, that it would take 9 days, 1 to 28 days, 7 days, 7 days, and 11 weeks to complete Explore, Build, Mobilize, Quit, and Secure, respectively. However, actual participant behavior demonstrated longer durations spent in some stages (Mobilize through Secure), and the study was designed to allow for this type of participant behavior. This flexibility is valuable in that it mirrors how Pivot will be available to and used by its initial intended users (employees of self-insured employers or employers with wellness programs). Nonetheless, this approach is different from the more traditional and rigid 30- or 60-day assessments that are linked directly to enrollment date, and this difference is worth acknowledging.

### Conclusions

Pivot is a comprehensive digital smoking program, which combines proven cessation principles derived from the USCPG with the innovation of a mobile CO breath sensor and in-app text-based human coaching. In this initial assessment, Pivot was shown to be engaging and quit rates were aligned with those in the peer-reviewed literature. This was true in both individuals who were and were not ready to quit in the next 30 days, with comparable retention and cessation outcomes between the 2 groups. Pivot leverages accessible and nearly ubiquitous smartphone technology and engages individuals along the entire spectrum of readiness to quit—something few programs have done to date. The hope is this combination will translate to increased reach; these initial results are encouraging. Looking forward, this study informs the future development of Pivot. Next steps include implementing and evaluating refinements to the program based on present learnings and further evaluation via a randomized controlled trial.

## Abbreviations

ACT: Acceptance and Commitment Therapy
CO: carbon monoxide
CPD: cigarettes per day
FDA: Food and Drug Administration
ITT: intention-to-treat
PPA: point prevalence abstinence
USCPG: United States Clinical Practice Guideline
